# A Crop Classification Method Integrating GF-3 PolSAR and Sentinel-2A Optical Data in the Dongting Lake Basin

**DOI:** 10.3390/s18093139

**Published:** 2018-09-17

**Authors:** Han Gao, Changcheng Wang, Guanya Wang, Jianjun Zhu, Yuqi Tang, Peng Shen, Ziwei Zhu

**Affiliations:** 1 School of Geosciences and Info-Physics, Central South University, Changsha 410083, China; dawnhan314@csu.edu.cn (H.G.); wangguanya@csu.edu.cn (G.W.); zjj@csu.edu.cn (J.Z.); yqtang@csu.edu.cn (Y.T.); shen-peng@csu.edu.cn (P.S.); zhumyrtle@csu.edu.cn (Z.Z.); 2 Key Laboratory of Metallogenic Prediction of Nonferrous Metals and Geological Environment Monitoring, Ministry of Education, Central South University, Changsha 410083, China

**Keywords:** GF-3, PolSAR data, Sentinel-2A, optical data, data integration, crop classification, Dongting lake basin

## Abstract

With the increasing of satellite sensors, more available multi-source data can be used for large-scale high-precision crop classification. Both polarimetric synthetic aperture radar (PolSAR) and multi-spectral optical data have been widely used for classification. However, it is difficult to combine the covariance matrix of PolSAR data with the spectral bands of optical data. Using Hoekman’s method, this study solves the above problems by transforming the covariance matrix to an intensity vector that includes multiple intensity values on different polarization basis. In order to reduce the features redundancy, the principal component analysis (PCA) algorithm is adopted to select some useful polarimetric and optical features. In this study, the PolSAR data acquired by satellite Gaofen-3 (GF-3) on 19 July 2017 and the optical data acquired by Sentinel-2A on 17 July 2017 over the Dongting lake basin are selected for the validation experiment. The results show that the full feature integration method proposed in this study achieves an overall classification accuracy of 85.27%, higher than that of the single dataset method or some other feature integration modes.

## 1. Introduction

As for the demand of large-scale and high-efficiency crop mapping, remote sensing technology can substitute for the traditional field measurement and it can observe the same area many times in a short revisit time. Nowadays, optical data and polarimetric synthetic aperture radar (PolSAR) data are often used for crops’ monitoring and the integration of multi-source data sets can help to achieve high-precision classification results. However, in the integrated classification, some effective features extracted from data of different sensors cannot be used at the same time, so that the potential of integrated datasets cannot be fully explored. Particularly, the covariance matrix of PolSAR data is difficult to be combined with multi-spectral optical data for classification. Considering the covariance matrix contains rich polarimetric information, this paper applies Hoekman’s method [1], the matrix can be transformed to an intensity vector, detailed in Section 3.2. Such intensity vector has nine bands, denoting the intensity values on different polarization bases, which has the similar data structure with the spectral bands of optical data, so it is easy to combine these two kinds of information. In addition, some other useful features are extracted, including the polarimetric features, as the radar vegetation index (RVI) and the decomposed Yamguichi four components, as well as some optical features as the normalized difference vegetation index (NDVI) and the information entropy describing the texture information. The spectral characteristics in the optical data are mainly used to indicate the changes in the moisture and chlorophyll content of the crop leaves [2,3]. In the PolSAR data, the backscatter information of the multiple polarimetric channels are used to describe the structure, orientation distribution and dielectric constant characteristics of crops [4,5,6,7,8,9]. Generally speaking, the optical and PolSAR data can characterize different properties of crops. These two data are mutually independent and complementary to each other. There are some methods developed for using each of these data set for crop classification, including the PolSAR classification methods [10,11,12,13,14,15,16,17,18,19] and the optical classification methods [20,21,22,23,24]. However, the limited kinds of observation measurements by single type of satellite is hard to fully represent the characteristics of targets and the combination of multi-source data can be used for crop classification [25,26,27,28,29,30,31,32].

Nowadays, data fusion and data integration are two common combination modes of multi-source data. Particularly, compared with the data integration methods, there are more data fusion methods, as PCA fusion method [33,34], Brovey fusion method [35,36], Gram-Schmidt transform fusion method [37,38], wavelet transform method [39,40,41,42]. However, the dimension of feature sets extracted in data fusion is generally three, corresponding to the RGB channels for visual representation. Due to the number of feature sets extracted in data fusion is fewer than the data integration, the classification accuracy of data fusion method is lower [43]. So, the data integration is applied in the classification.

Furthermore, the extracted feature sets can be applied into crop classification. Available classification algorithms include the maximum likelihood algorithm [44], the support vector machine (SVM) [29,45], the neural network [46], the deep learning algorithm [47]. Among which, the maximum likelihood algorithm is based on the probability distribution of the characteristics of feature sets, which is simple and easy to be operated. But its classification accuracy is low, because the selected distribution model may not be suitable for all terrain types. Other three methods all belong to machine learning algorithms, which use training samples for iterative learning. And the classification rules can be generated to identify the unknown objects. The neural network and deep learning algorithm require a large number of training samples and the training process is time-consuming, caused by the high model complexity. Whereas, the SVM algorithm is to convert the feature sets into high dimensional space through a kernel function and to generate a classification plane. It needs only a few training samples and has low modeling complexity and good usability. So, it has been applied in many cases of classification and recognition of objects.

The paper is organized as follows. Section 2 illustrates the study area and datasets. Section 3 describes the main detailed steps of the proposed method, including data preprocessing, feature extraction and integration and SVM classification. Section 4 presents the experimental results. Section 5 makes some detailed discussions for the results. Finally, we draw some conclusions in Section 6.

## 2. Study Area and Dataset

**Figure 1:**
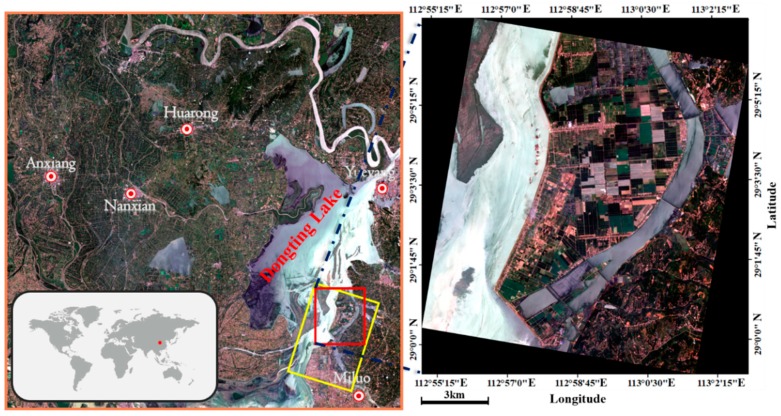
The location of the study area and the used data coverage, the yellow and orange rectangle denotes the GF-3 PolSAR data and the Sentinel-2A optical data, respectively. The red rectangles outline the experimental area.

The study area is located in the southeastern Dongting Lake basin, Hunan, China (Figure 1). The main crops there are rice, watermelon and lotus. With the steady stream of irrigation support from Dongting Lake, there grows the single-season rice (Rice1) and the two-season rice (Rice2). We selected the GF-3 polarimetric SAR data acquired on 19 July 2017 and Sentinel-2A optical data on 17 July 2017, for crop classification. The specific imaging parameters of GF-3 data and Sentinel-2A data are shown in Table 1 and Table 2, respectively. Hereon, the research based on the satellite GF3 can expand the application of GF-3 data in agriculture. As the first C-band synthetic aperture radar (SAR) satellite in China, it owns 12 imaging modes with the highest spatial resolution of 1 m [48]. GF-3 satellite is able to monitor the ocean and the land under any weather conditions. Moreover, its unique left and right side looking modes improve its ability of quick response to the emergence of disasters.

**Figure 2:**
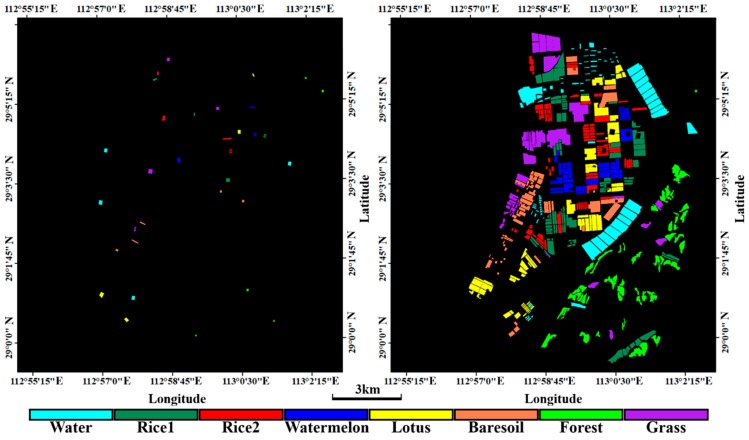
The training (**left**) and testing (**right**) samples in the study area.

We collected the crop information through an in-situ survey. We kept a record for crop types and their growth stages. The crop types were identified through the regional agricultural expertise and farmers. Finally, the training samples and testing samples were separately selected (Figure 2) according to the basic sampling principle [49,50] and the detailed information of samples are listed in Table 3.

## 3. Methodology

**Figure 3:**
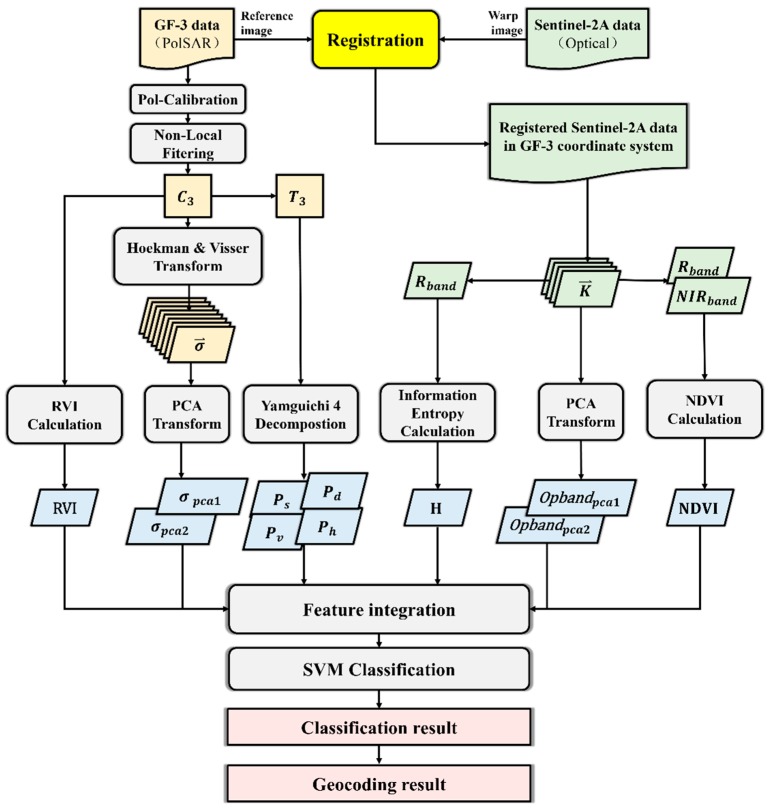
The flowchart of the proposed method.

The proposed method includes the following steps: data preprocessing, feature extraction and integration, SVM classification. The flowchart of the proposed method is shown in Figure 3.

### 3.1. Data Preprocessing

In order to make the extracted features better used for classification, the careful data preprocessing is necessary. Firstly, the GF-3 data is polarimetric calibrated. Specifically, the backscattering amplitude information on different polarization channels should be corrected according to the calibration constants in the header file. Then, the polarimetric coherency matrix *T*_3_ is generated and the Non-Local filtering is used to reduce the speckle noise [51,52]. Finally, the area of interest is selected for subsequent experiments. As for the Sentinel-2A data, there are 13 bands, of which the selected four bands are commonly used for classification, including red (R), green (G), blue (B) and near infrared (NIR) bands.

**Figure 4:**
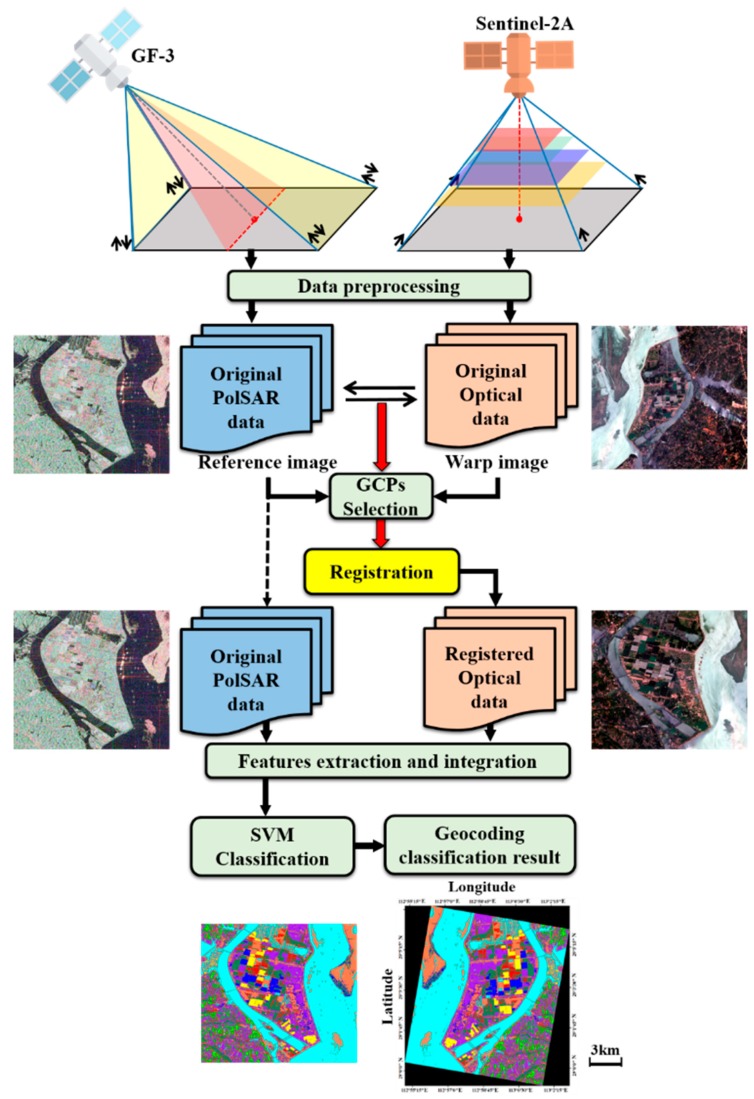
The registration process of the proposed method.

Then the two data are registered into the same coordinate system for extracting and integrating features. Because the SAR acquisition is side looking, which is different from the central projection of optical data, the original optical data is registered into the SAR coordinate system for keeping target’s backscattering characteristics. Details are shown in Figure 4. We choose the ground control points (GCPs) and then register data sets based on corresponding GCPs. Since the study area has a flat terrain, the SAR data has no obvious foreshortening, layover and shadow. So, the registration method based on the GCPs can achieve a high registered accuracy. At last, the optical data is cut into the interested area the same as GF-3 data.

### 3.2. Feature Extraction and Integration

To fully characterize different crops, we extract the backscattering intensity, backscattering type, canopy vegetation index from the GF-3 data and the spectral characteristics, spatial texture, canopy vegetation index from Sentinel-2A data. Since the intensity information is the most direct representation of the backscattering of radar waves in ground objects, it is extracted firstly.

We use the method proposed by Hoekman in 2003 [1] to transform elements of covariance matrix *C*_3_ into multi-channel intensity vectors. Matrix *B* can be used to convert the elements of matrix *C*_3_ into an intensity vector $\vec{P}$, which can represent the backscattering intensity of crops in different polarimetric channels. The equation is shown specifically as follows:

(1) $$\left[\begin{array}{c} \langle {\left|S_{HH}\right|}^{2}\rangle \\ \langle {\left|S_{VV}\right|}^{2}\rangle \\ \langle {\left|S_{HV}\right|}^{2}\rangle \\ Re\left[\langle S_{HH}S_{VV}^{*}\rangle \right]\\ Im\left[\langle S_{HH}S_{VV}^{*}\rangle \right]\\ Re\left[\langle S_{HH}S_{HV}^{*}\rangle \right]\\ Im\left[\langle S_{HH}S_{HV}^{*}\rangle \right]\\ Re\left[\langle S_{HV}S_{VV}^{*}\rangle \right]\\ Im\left[\langle S_{HV}S_{VV}^{*}\rangle \right] \end{array}\right]=B\vec{P}=B{\left[\begin{array}{c} DN_{hh}\\ DN_{vv}\\ DN_{++45}\\ DN_{--45}\\ DN_{ll}\\ DN_{rr}\\ DN_{h+45}\\ DN_{hl}\\ DN_{+45l} \end{array}\right]}_{9\times 1}$$

(2) $$B=\frac{1}{4\pi }{\left[\begin{array}{ccccccccc} 1 & 0 & 0 & 0 & 0 & 0 & 0 & 0 & 0\\ 0 & 1 & 0 & 0 & 0 & 0 & 0 & 0 & 0\\ -\frac{1}{4} & -\frac{1}{4} & +\frac{1}{4} & +\frac{1}{4} & +\frac{1}{4} & +\frac{1}{4} & 0 & 0 & 0\\ 0 & 0 & +\frac{1}{2} & +\frac{1}{2} & -\frac{1}{2} & -\frac{1}{2} & 0 & 0 & 0\\ +\frac{1}{4} & +\frac{1}{4} & +\frac{3}{4} & -\frac{1}{4} & +\frac{3}{4} & -\frac{1}{4} & 0 & 0 & -2\\ -\frac{3}{8} & +\frac{1}{8} & -\frac{1}{8} & -\frac{1}{8} & -\frac{1}{8} & -\frac{1}{8} & 1 & 0 & 0\\ +\frac{3}{8} & -\frac{1}{8} & +\frac{1}{8} & +\frac{1}{8} & +\frac{1}{8} & +\frac{1}{8} & 0 & -1 & 0\\ +\frac{3}{8} & -\frac{1}{8} & +\frac{5}{8} & -\frac{3}{8} & +\frac{1}{8} & +\frac{1}{8} & -1 & 0 & 0\\ -\frac{3}{8} & +\frac{1}{8} & -\frac{1}{8} & -\frac{1}{8} & -\frac{5}{8} & +\frac{3}{8} & 0 & 1 & 0 \end{array}\right]}_{9\times 9}$$

where DN denotes the intensity value and the subscripts denote the received and transmitted polarization bases: horizontal (*h*), vertical (*v*), left circular (*l*), right circular (*r*), 45° linear (+ or +45) and −45° linear (− or −45).

It is worth noting that the backscattering intensity often contains a number of large magnitude values. For the normalization during the data combination, we transform the original intensity into the intensity with backscattering coefficient format (dB) by

(3) $$\vec{\sigma }=10{log}_{10}\left(\vec{P}\right)$$

(4) $$\vec{\sigma }={\left[\begin{array}{c} \sigma_{hh}\\ \sigma_{vv}\\ \sigma_{++45}\\ \sigma_{--45}\\ \sigma_{ll}\\ \sigma_{rr}\\ \sigma_{h+45}\\ \sigma_{hl}\\ \sigma_{+45l} \end{array}\right]}_{9\times 1}$$

where $\vec{\sigma }$ denotes the transformed intensity vector and its detailed values are presented in Formula (4). The subscripts in $\vec{\sigma }$ are the same with $\vec{P}$. Although, the backscattering intensity information can be characterized by $\vec{\sigma }$, the dimension of $\vec{\sigma }$ in multi-source data integration is large and will lead to data redundancy. Such redundancy will reduce the classification accuracy and computational efficiency. The principal component analysis (PCA) algorithm can pick out one or two main eigenvalues to replace the total eigenvector, so as to increase the classification accuracy and computational efficiency. In this paper, the sum of the first two principal components’ variance values accounts for 98% of the total, which can be used to substitute for eigenvector in the calculation. In addition, such two principal component features σ_pca1_ and σ_pca2_ are extracted.

As for the backscattering type information, the corresponding polarimetric characteristics can be extracted by the Y4R decomposition method which is proposed by Yamguichi in 2005 [53]. On the basis of the classical Freeman three-component decomposition, the Y4R decomposition method further considers the helix scattering mechanism, which makes the backscattering types of polarimetric decomposition closer to the real situation, so that the Y4R method has been widely used for PolSAR image classification.

(5) $$Span={\left|S_{HH}\right|}^{2}+2{\left|S_{HV}\right|}^{2}+{\left|S_{VV}\right|}^{2}=P_{s}+P_{d}+P_{v}+P_{c}$$

(6) $$P_{s}=f_{s}\left(1+{\left|\beta \right|}^{2}\right)$$

(7) $$P_{d}=f_{d}\left(1+{\left|\alpha \right|}^{2}\right)$$

(8) $$P_{v}=f_{v}$$

(9) $$P_{c}=f_{c}$$

where $P_{s}$, $P_{d}$, $P_{v}$ and $P_{c}$ represents the scattering intensity of surface scattering, double scattering, volume scattering and helix scattering, respectively, $f_{s}$, $f_{d}$, $f_{v}$ and $f_{c}$ are the surface, double-bounce, volume, helix scattering contributions to ${\left|S_{VV}\right|}^{2}$, α and β denote the reals.

RVI extracted from PolSAR data can be used as the canopy vegetation index [54] and it applies the power of different polarimetric channels to reflect the canopy vegetation characteristics of different phenological stages. The greater the power, the closer the crop canopy is to the forest canopy.

(10) $$\mathrm{RVI}=\frac{8\langle {\left|S_{HV}\right|}^{2}\rangle }{\langle {\left|S_{HH}\right|}^{2}\rangle +\langle {\left|S_{VV}\right|}^{2}\rangle +2\langle {\left|S_{HV}\right|}^{2}\rangle }$$

The characteristics of crop spectral information, spatial texture information and canopy vegetation index are extracted from the Sentinel-2A optical data. Multi-spectral information is more sensitive to moisture and the chlorophyll component of crop leaves, which can be used to identify the crop species. In this paper, four common spectral bands (R, G, B, NIR) are extracted to characterize the spectral information of crops and their corresponding feature vectors are also transformed by PCA algorithm. The first two principal components Opband_pca1_ and Opband_pca2_ are extracted, of which sum can contribute 99% of the overall variance of eigenvector.

Then the information entropy H of image on the red (R) band is used to characterize the spatial texture information of crops. The information entropy is an indicator of uncertainty measurement. The greater the value, the higher the uncertainty [55]. As for the image on single spectral band, the uncertainty is often determined by the richness of texture. The richer the texture information, the higher the uncertainty.

At last, the normalized difference vegetation index (NDVI) is calculated from red and near infrared band images by Equation (11). NDVI is used to characterize the canopy properties of different crops, especially the changes of canopy density and biomass.

(11) NDVI=(NIR−R)/(NIR+R)

**Figure 5:**
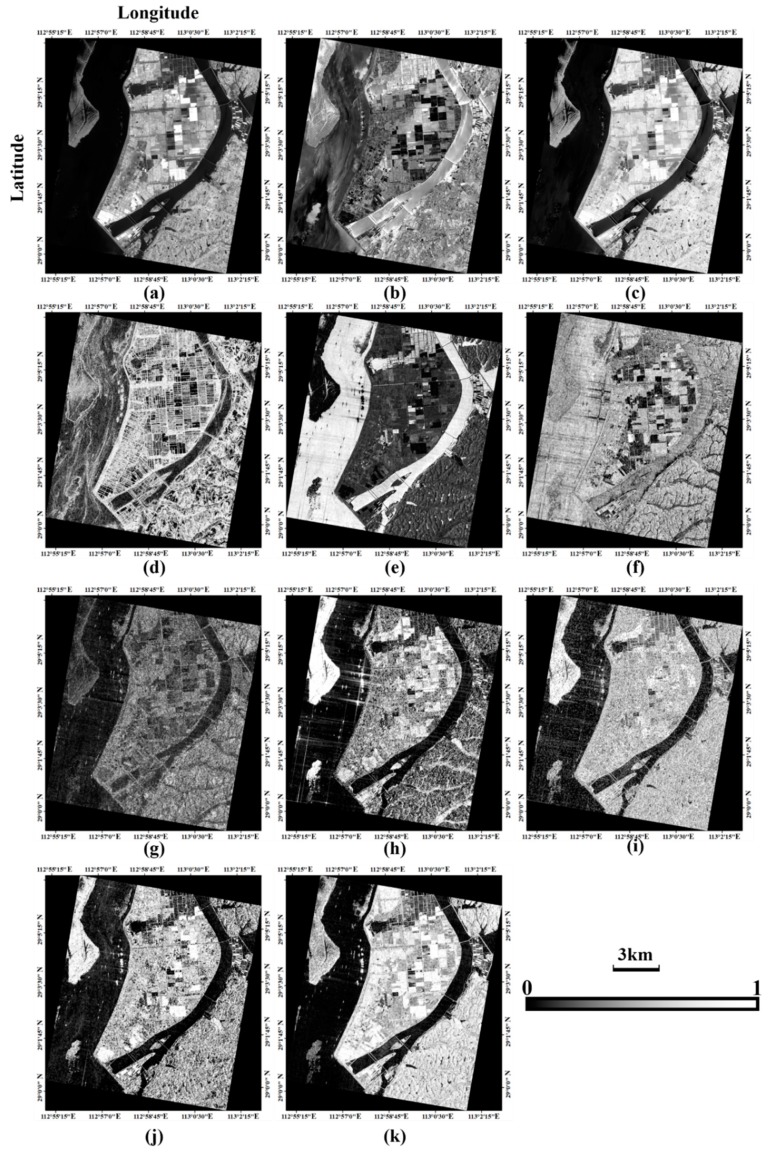
The features normalized to the range of [0,1]. (**a**–**d**) Opband_pca1_, Opband_pca2_, NDVI and the information entropy H extracted from the Sentinel-2A data; (**e**–**k**) σ_pca1_, σ_pca2_, RVI, *P_s_*, *P_d_*, *P_h_* and *P_v_* extracted from the GF-3 PolSAR data.

Then the extracted features should be integrated before the SVM classification. In order to eliminate the effects of different features’ scale, this paper normalizes all these features’ range to (0~1). As shown in Figure 5, the imaging characteristics between PolSAR data and optical data are obviously different. The features obtained by such two kinds of data are independent and complementary to each other.

### 3.3. SVM Classification

Based on the integrated features, the support vector machine (SVM) method is applied to crop classification. The SVM classifier is an excellent two-class classification model, which can use the kernel function to map the multi-dimensional feature sets into higher dimensional space, to construct the classification plane and distinguish different categories. This method can efficiently get high-precision classification results with a few training samples. The SVM classifier has been successfully applied in many aspects, such as land use classification mapping, data mining. The kernel function adopted in this paper is the radial basis function (RBF), which can solve the linear non-separable problem in SVM classification by nonlinear mapping and it has only several parameters and low model complexity. After the SVM classification, the results with the SAR coordinate system will be transformed into the geographic coordinate system.

## 4. Experimental Results

As shown in Table 4, the overall classification accuracy is 85.27% and the Kappa coefficient is 0.8306. As for the misclassification condition, the accuracy of water, lotus pond and vegetation has even reached 96% and that of the single-season rice, watermelon greenhouse, bare soil and grassland also reaches 80%. However, the misclassification rate of two-season rice is even higher than 54%. This is because the two-season rice has similar spectral characteristics as the single-season rice and vegetation. The omission rates of water, watermelon greenhouse and lotus pond are lower than 10% and that of bare soil and grassland is also lower than 20%. Besides, the omission rates of two kinds of rice are higher than the above five species, around 25%. While the omission rates of the vegetation are both over 30%. Although PolSAR can distinguish rice in different growing seasons, the classification accuracy is low, since there are nearly 1/4 of the two-season rice was misclassified as single-season rice. This could be resulted from the small number of available data. If the multi-temporal images are available, such two kinds of rice could be distinguished with the temporal information. And the omitted vegetation pixels here are mainly classified as the two-season rice and grassland. The reason is that the vegetation mostly grows in undulated mountains, where the speckle noise is stronger in PolSAR images and reduce the classification accuracy.

## 5. Discussion

### 5.1. Comparison with Different Datasets

**Figure 6:**
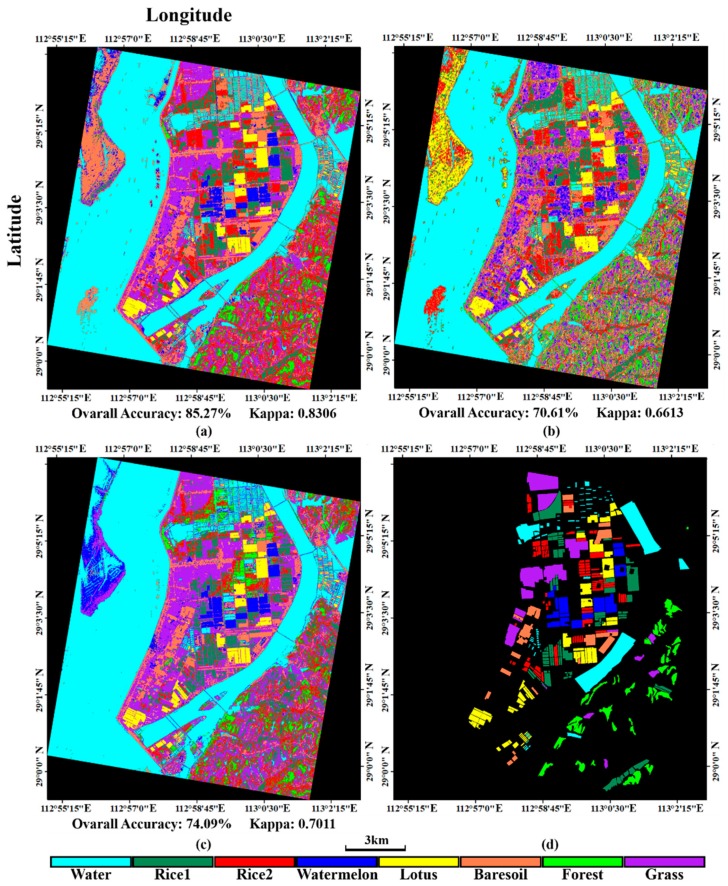
The classification results generated from (**a**) the integrated data (**b**) the GF-3 data and (**c**) the Sentinel-2A data; (**d**) the testing sample.

**Figure 7:**
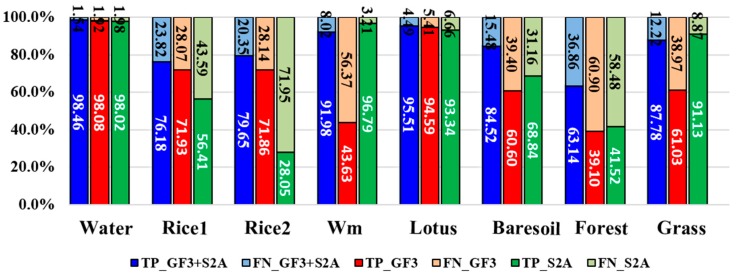
True positive (TP) rates and false negative (FN) rates of different land covers from different datasets. Wm means watermelon.

**Figure 8:**
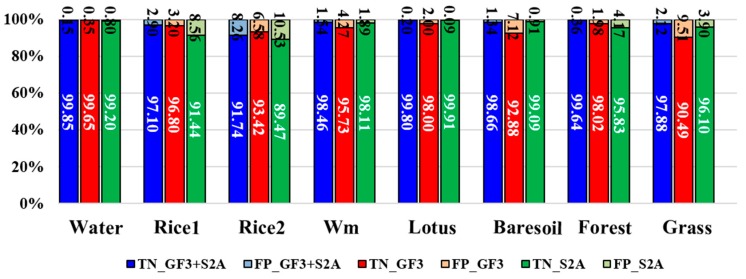
True negative (TN) rates and false positive (FP) rates of different plants from different datasets. Wm means watermelon.

To validate the proposed full feature integration method, this section compares the results generated from the integrated data and that from single GF-3 data as well as from the single Sentinel-2A data (Figure 6). We also assessed the classification accuracies. The evaluated indicators are the rates of true positive (TP), false negative (FN), true negative (TN) and false positive (FP). These indicators can fairly evaluate result on each class no matter how many samples are used [56]. We present these indicators by histograms. The sum of TP’s rate and FN’s rate equals to 1, which can be shown in one bar of the histogram (Figure 7). And the case is the same for the TN’s rate and FP’s rate (Figure 8). It can be seen that the overall classification accuracy of the integrated data is the highest, followed by the single optical data, then the single PolSAR data. The GF-3 PolSAR data alone can distinguish single-season rice from two-season rice but it will misclassify bare soil, grassland and watermelon greenhouse mainly with the surface scattering. While the Sentinel-2A data alone performs oppositely to GF-3 PolSAR data. It shows better classification ability for bare soil, grassland and watermelon greenhouse, because the spectral information of these three land covers varies greatly. But it cannot classify the single-season rice and two-season rice as well as the GF-3 data, providing a classification accuracy of two-season rice of as low as 28%. The proposed integration method takes the advantages of both two data, so the results have the highest classification accuracy.

### 5.2. Comparison with Different Feature Integration Modes

**Figure 9:**
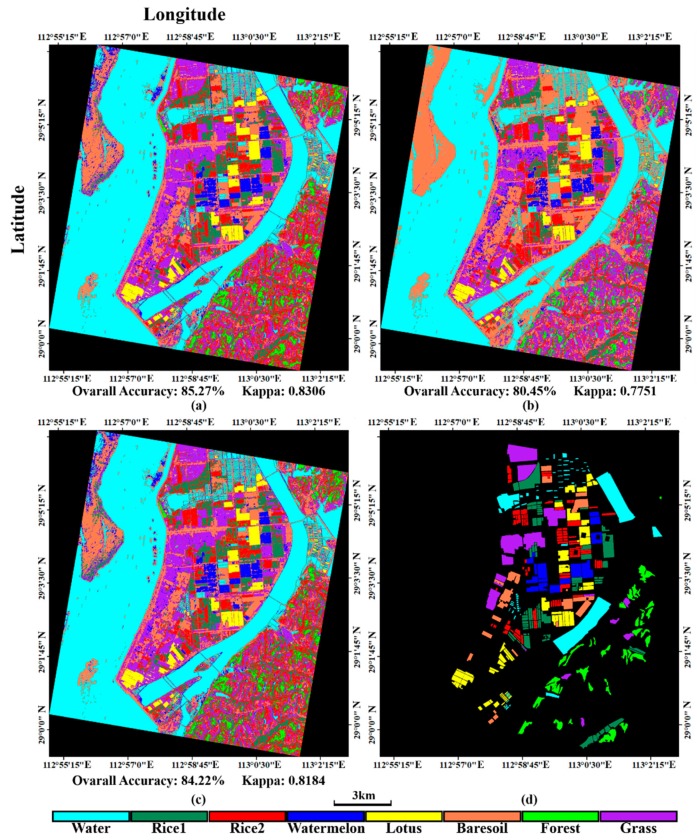
The classification results of (**a**) integration with of all features of dataset; (**b**) GF-3 (7 bands) + S2A (2 bands) and (**c**) denotes GF-3 (5 bands) + S2A (4 bands); (**d**) the testing sample.

**Figure 10:**
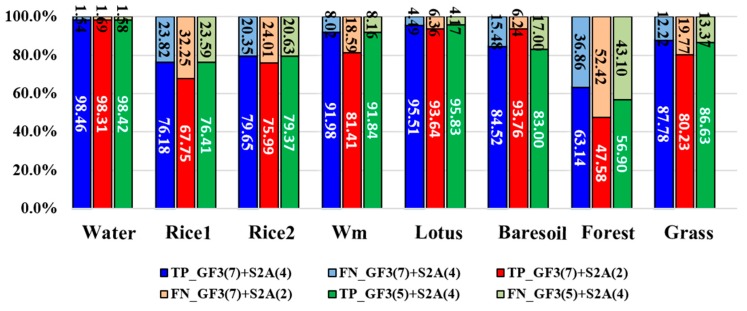
The true positive (TP) rates and the false negative (FN) rates of different land covers generated from different combination of features. Wm means watermelon.

**Figure 11:**
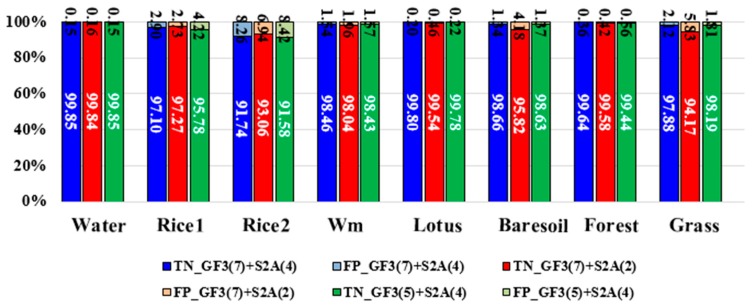
The true negative (TN) rates and the false positive (FP) rates of different land covers generated from different combination of features. Wm means watermelon.

This section aims to validate the advantage of full feature integration proposed by this paper. Traditional data fusion methods think that both the intensity values of SAR data and the spectral information of optical data into classification at the same time, leading to data redundancy. But the intensity of SAR data is different from the spectral information of optical data. The former denotes the backscattering characteristics, whereas the latter denotes the reflection of sunlight. The classification results under different feature integration modes will be discussed and the details are shown in Table 5. In this study, we used three feature integration modes, including (1) GF-3 features (σ_pca1_, σ_pca2_, RVI, *P_s_*, *P_d_*, *P_h_* and *P_v_*) + Sentinel-2A features (Opband_pca1_, Opband_pca2_, NDVI and H); (2) GF-3 features (σ_pca1_, σ_pca2_, RVI, *P_s_*, *P_d_*, *P_h_* and *P_v_*) + Sentinel-2A features (NDVI and H); (3) GF-3 features (RVI, *P_s_*, *P_d_*, *P_h_* and *P_v_*) + Sentinel-2A features (Opband_pca1_, Opband_pca2_, NDVI and H). The classification results are shown in Figure 9 and the accuracy assessments are shown in Figure 10 and Figure 11. It can be concluded that, the full feature integration method has achieved the highest overall classification accuracy and larger Kappa coefficient. It is mainly owing to the improvement of the classification accuracy of vegetation and grassland. And the involvement of more features makes the classification more accurate and stable. In addition, it can be seen that when the PolSAR features are more involved (GF-3 (7 bands) + S2A (2 bands)), the classification accuracy of single-season rice and two-season rice is increased. However, when more optical features are involved (GF-3 (5 bands) + S2A (4 bands)), the classification accuracy of bare soil and watermelon greenhouse is improved. So, this conclusion is consistent with that of last section. To sum up, the full feature integration method proposed in this paper can get a higher classification accuracy.

### 5.3. Classification Ability of $\vec{\sigma }$

**Figure 12:**
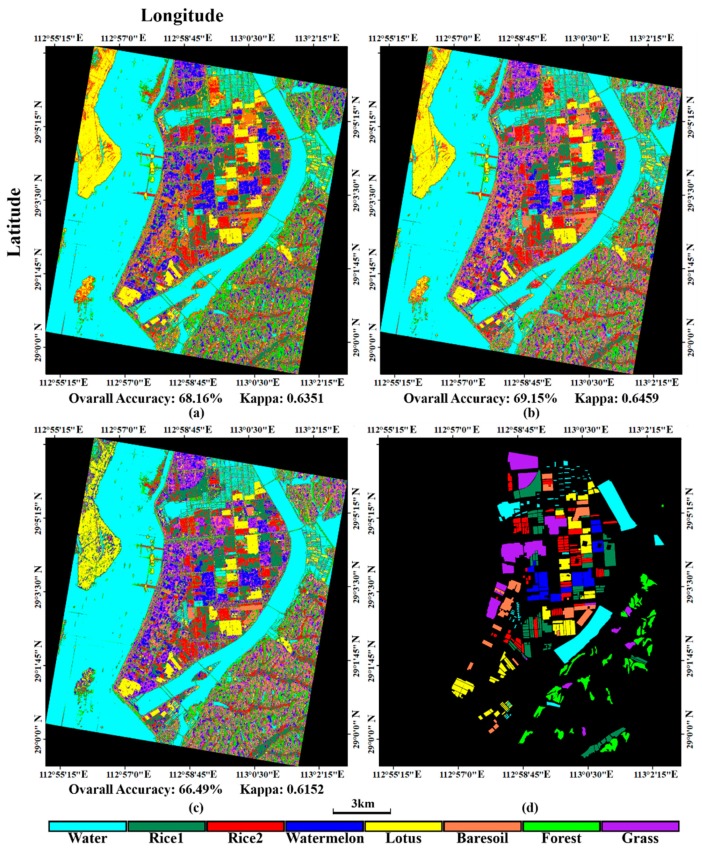
The classification results of different polarimetric classification methods. (**a**) the Wishart supervised classification with $C_{3}$; (**b**) the SVM classification with $\vec{\sigma }$ and (**c**) the SVM classification with the first two PCA components of $\vec{\sigma }$; (**d**) the testing sample.

**Figure 13:**
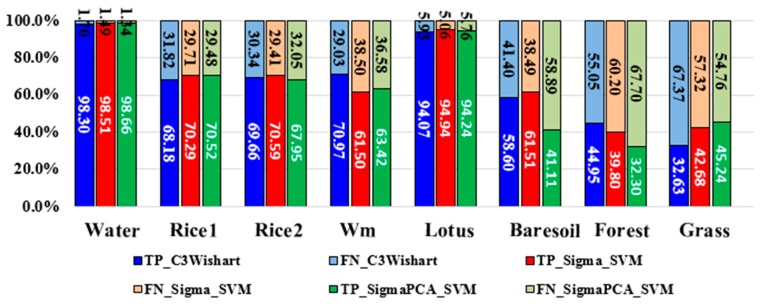
The true positive (TP) rates and the false negative (FN) rates of different polarimetric classification methods. Wm means watermelon.

**Figure 14:**
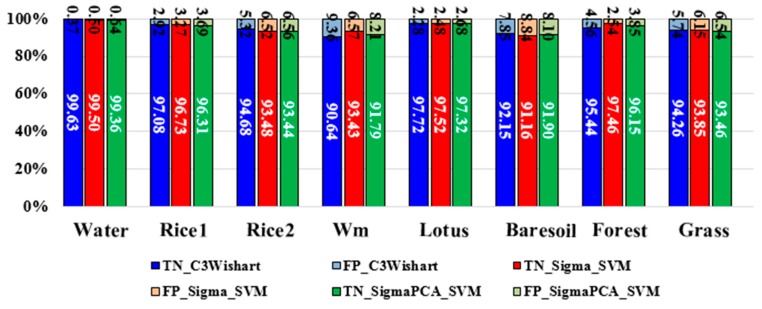
The true negative (TN) rates and the false positive (FP) rates of different polarimetric classification methods. Wm means watermelon.

The Wishart supervised classification based on the covariance matrix $C_{3}$ or the coherency matrix $T_{3}$ has be widely used. In this study, we substituted the intensity vector $\vec{\sigma }$ for covariance matrix to adapt to the SVM classifier. Input variables of the SVM classifier should be multiple independent bands. Hoekman has proved that the intensity vector $\vec{\sigma }$ can represent the full polarimetric target characteristics by a covariance matrix [1] and $\vec{\sigma }$ is more suitable to crop classification, because it can describe the biophysical parameter variations of crops. To clarify this point, we compare three polarimetric classification methods, including (1) Wishart supervised classification with $C_{3}$, (2) SVM classification with $\vec{\sigma }$ and (3) SVM classification with the first two PCA components of $\vec{\sigma }$. The results are presented in Figure 12. As the figure shows, the SVM classification with $\vec{\sigma }$ has the highest overall accuracy and kappa coefficient in all methods. We also calculated the rates of TP, FN, TN and FP and made a comparison (Figure 13 and Figure 14). The comparison shows that the SVM method with $\vec{\sigma }\;$ performs better than the Wishart supervised method in most land covers but the Wishart method has the best performance in the watermelon greenhouse and the forest region among these three methods. The crop classification results of the SVM classification with $\vec{\sigma }$ has the highest accuracy, verifying Hoekman’s theory that $\vec{\sigma }$ is more suitable to describe crops. And for the crops, the first two PCA components of $\vec{\sigma }\;$ can achieve similar classification results as the whole $\vec{\sigma }$. We can conclude that the intensity vector and its PCA components can be successfully applied into the polarimetric classification and get better results than the Wishart supervised classification in most crop cases.

## 6. Conclusions

The GF-3 PolSAR data is sensitive to the change of morphological structure during crop growth, whereas the Sentinel-2A optical data can show the change of moisture and chlorophyll content in crop leaves well. Integrating such two kinds of data can improve the accuracy of crop classification. However, some useful features cannot be used in the classification at the same time. Particularly, the covariance matrix of PolSAR data is hard to be combined with the spectral bands of optical data. To solve this problem, we used the Hoekman’s method to transform the covariance matrix to an intensity vector. The PCA algorithm was applied to reduce the redundancy of feature sets. Then, the training samples were selected to do the SVM classification. The classification accuracy of the proposed method is higher than that of single data set method and other two feature integration modes and the intensity vector has a better performance than the covariance matrix for crop classification. In total, full feature integration method proposed by this paper is suitable for crop classification and can effectively improve the classification accuracy. Furthermore, this paper expands the application of GF-3 satellite in agriculture, proving the great potential in monitoring crops.

## Figures and Tables

**Table 1:** Main imaging parameters of GF-3 satellite.

Item	Parameter
Polarization mode	HH, HV, VH and VV
Chirp Bandwidth (MHz)	40
Centre frequency (GHz)	5.400012
Band	C-band
Range pixel spacing (m)	2.248443
Azimuth pixel spacing (m)	4.733369
Acquisition Type	Stripmap (QPSI)
Start time	2017-07-19, 22:26:57.615189
Stop time	2017-07-19, 22:27:01.799853
Incidence angle	38.16°

**Table 2:** Main imaging parameters of Sentinel-2A satellite.

Item	Parameter
Swath (km)	290
Acquisition time	2017-07-17, 11:05:41.26
Spectral bands	R (Band 4), G (Band 3), B (Band 2), NIR (Band 8)
Centre Wavelength (nm)	R (665), G (560), B (490), NIR (842)
Bandwidth (nm)	R (30), G (35), B (65), NIR (115)
Spatial Resolution (m)	R (10), G (10), B (10), NIR ( 0)
Reference Radiances *L_ref_* (W m^−2^ sr^−1^ µm^−1^)	R (108), G (128), B (128), NIR (103)
Signal-to-Noise Ratios @ *L_ref_*	R (142), G (168), B (154), NIR (174)

**Table 3:** Field data collected for classification training and testing.

Land Cover	Training Samples		Testing Samples	
	Number of Pixels	Number of Plots	Number of Pixels	Number of Plots
Water	5118	4	241,174	86
Rice (single-season)	3305	5	199,891	81
Rice (two-season)	3572	5	122,269	91
Watermelon	2679	4	106,678	52
Lotus	4193	4	188,068	56
Bare soil	2841	5	134,727	55
Forest	1890	5	168,832	52
Grass	4336	4	208,945	54

**Table 4:** Classification accuracy assessment of the integrated dataset.

Pixels	Water	Rice1	Rice2	Wm	Lotus	Bare Soil	Forest	Grass	UA (%)
Water	237,449	0	0	0	35	1052	249	377	99.28
Rice1	5	152,273	21,550	9	3014	882	6381	1965	81.83
Rice2	124	44,750	97,382	13	392	1108	41935	13133	48.98
Wm	624	34	3	98,113	68	13364	361	3812	84.30
Lotus	0	104	13	0	179,632	0	270	1790	98.80
Bare soil	2732	591	98	8074	92	113,877	384	3540	88.01
Forest	125	342	2715	0	50	81	106,606	910	96.19
Grass	115	1797	508	463	4785	4363	12642	183,418	88.14
PA (%)	98.46	76.18	79.65	91.98	95.51	84.52	63.14	87.78	
Overall Accuracy (%)			85.2745		Kappa coefficient			0.8306	

Note: Wm denotes “Watermelon.” The user’s accuracy (UA) indicates the misclassification condition, while the producer’s accuracy (PA) indicates the omission condition.

**Table 5:** Details on different feature integration modes.

Feature Integration Mode	GF-3 Features	Sentinel-2A Features
GF-3 (7 bands) + S2A (4 bands)	σ_pca1_, σ_pca2_, RVI, *P_s_*, *P_d_*, *P_h_* and *P_v_*.	Opband_pca1_, Opband_pca2_, NDVI and H
GF-3 (7 bands) + S2A (2 bands)	σ_pca1_, σ_pca2_, RVI, *P_s_*, *P_d_*, *P_h_* and *P_v_*.	NDVI and H
GF-3 (5 bands) + S2A (4 bands)	RVI, *P_s_*, *P_d_*, *P_h_* and *P_v_*.	Opband_pca1_, Opband_pca2_, NDVI and H
